# Assessing Orthogonality in Gene-Environment Interaction Studies Using Polygenic Indices

**DOI:** 10.1007/s10519-025-10248-8

**Published:** 2026-01-09

**Authors:** Eric A. W. Slob, Dilnoza Muslimova, Cornelius A. Rietveld

**Affiliations:** 1 Erasmus School of Social and Behavioural Sciences, Erasmus University Rotterdam, Burgemeester Oudlaan 50, 3062PA Rotterdam, The Netherlands; 2 Erasmus School of Economics, Erasmus University Rotterdam, Burgemeester Oudlaan 50, 3062 PA Rotterdam, The Netherlands

**Keywords:** Gene-environment interactions, Orthogonality, Polygenic index

## Abstract

Gene-environment interaction (G×E) studies analyze how environmental conditions cushion or exacerbate differences in genetic endowments. A gene-environment correlation (*rGE*) between the polygenic index (PGI) and the environmental condition employed in these G×E studies could bias the estimation of the interaction effect. In this brief report, we discuss the limitations of the commonplace correlation-based test used to verify the orthogonality of the PGI and the environment, and propose to complement it with an additional assessment of the genetic correlation between the phenotype corresponding to the PGI of interest and the environmental condition in the G×E analysis sample using bivariate GREML. Our proposed test is straightforward to perform with the data typically available to G×E researchers, and bypasses that the PGI reflects the environmental conditions of the training sample used to calibrate it. Using UK Biobank data, we provide empirical illustrations covering three environmental conditions relevant for educational attainment. We confirm the orthogonality of the Raising of School Leave Age 1972 educational reform and of gender, although gender did not pass the correlation-based test. However, birth district social class and the genetic propensity for educational attainment appear to be intrinsically intertwined.

**Supplementary Information:**

The online version contains supplementary material available at 10.1007/s10519-025-10248-8.

## Introduction

Heritability studies show that genes significantly contribute to inequalities in health and other important life outcomes such as socio-economic status (SES) (Polderman et al. 2015). Whether these genetic effects co-vary with or are independent of environmental conditions can be analyzed in so-called gene-environment interaction (G×E) studies (Wagner et al. 2013; Plomin 2014). While gene-environment interactions bring important insights on how environments can change the effect of genetic predispositions and vice versa, non-causal correlations between genes and the same environments can potentially bias the gene-environment interaction estimates. The severity of the bias in gene-environment interactions due to gene-environment correlations depends on the underlying mechanisms leading to this correlation. Simple multicollinearity would only inflate the standard errors without biasing the estimates. However, there are many instances when the gene-environment correlation is driven by more serious underlying issues, such as endogeneity, omitted variable bias, measurement error, and selection or collider bias. These issues are known to substantially bias the gene-environment interaction estimates (Biroli et al. 2025).

For example, Abdellaoui et al. (2019) report wide-spread gene-environment correlations in the UK Biobank, at least partly caused by selective migration across UK regions. This study illustrates that gene–environment correlations can be passive or active: a passive correlation occurs when parents provide both the genes and the environment that influence an outcome (for example, educated parents passing on both cognitive abilities and a stimulating home environment), whereas an active correlation arises when individuals seek out environments that match their genetically influenced preferences or abilities (as noted by Jencks 1980). In both cases, genetic selection into an environment would not only lead to a non-zero heritability estimate of the environment, but also to a genetic correlation with the outcome of interest. Therefore, to avoid bias in the G×E coefficient resulting from gene-environment correlation (*rGE*), the environmental condition in a G×E model needs to be orthogonal to the genetic propensity (Conley 2016; Biroli et al. 2025). Such orthogonal environmental conditions, for instance resulting from policy reforms or intervention discontinuities, are exogenously determined outside the model and independent of genetic predispositions. Besides theoretically motivating the exogeneity of the environment, a correlation-based test is usually suggested to probe the orthogonality of G and E (Biroli et al. 2025). For categorical environmental conditions (e.g., treated vs. non-treated), this test is equivalent to the comparison of the mean of the PGI distribution across the different environmental strata. While easy to perform, this test may fail short due to specific challenges that come with employing so-called polygenic indices (PGIs) in G×E studies.

Current G×E studies typically draw on PGIs rather than individual single nucleotide polymorphisms (SNPs) because of their larger explanatory power. PGIs are weighted combinations of multiple SNPs, and these weights are proportional to the strength of the relationship between a SNP and an outcome as estimated in a genome-wide association study (GWAS) in an independent training sample (Becker et al. 2021; Alemu et al. 2025). As a result, the GWAS weights reflect the environmental conditions of the GWAS training sample. This property affects the results of the correlation test between the PGI and the environment, which is conducted in the G×E analysis sample. Consider, for instance, that the GWAS training sample and G×E analysis sample are genetically identical. However, because of their birth year, half of the G×E analysis sample is exposed to a policy change that increases the compulsory years of schooling by one year. Examples of such a policy change include the “Raising of School Leave Age” (RoSLA) reform of 1972 in the United Kingdom as exploited in several recent UK Biobank-based (G×E) studies. Individuals with a low genetic propensity to achieve high education are most strongly affected by the policy change (Barcellos et al. 2018), as those individuals who would have dropped out after the earlier minimum leaving age (e.g., 15) now leave school when one year older (i.e., 16). This G×E interaction is not reflected in the GWAS weights in the training sample, because none of the individuals in the training sample was exposed to this policy reform. As a result, the correlation between the PGI and the environment in the G×E analysis sample will be insignificant. As long as individuals exposed to the reform are genetically similar to those not exposed, one can go ahead with the G×E analysis.

However, in the presence of wide-spread gene-environment correlations (Abdellaoui et al. 2019), such genetic similarity appears to be a strong assumption. Apart from population dynamics, such genetic differences are likely to be present, because genotyped samples are typically not representative for a particular population. For instance, Barcellos et al. (2018) show in their Appendix mild differences in the mean of the PGI for education attainment in their G×E analysis sample between those exposed and non-exposed to the RoSLA1972 reform. Moreover, while gender is a typical environmental factor exploited in G×E studies (e.g., Miao et al. 2025), participation bias in often-used genotyped samples causes sex-differentials in genetic analyses (Pirastu et al. 2021). The correlation-based test between the PGI and the environment in the G×E analysis sample may thus pick up differential exposure to (changing) environments. Weights obtained in GWAS training samples (which are typically meta-analyzed aggregates of smaller samples) to create a PGI may therefore not be suitable to test for orthogonality between the PGI and E. We therefore advocate the complementary value of a test of the orthogonality between G and E that is independent of GWAS weights in the GWAS discovery sample.

We propose using bivariate Genome-based Restricted Maximum Likelihood (GREML; Yang et al. 2010; Lee et al. 2012) estimation of the SNP-based genetic correlation $r_{g} \left( {Y,\,E} \right)$ between the outcome of the PGI, Y, and the environment, E, to test for the orthogonality of the genetic propensity for a particular outcome and the environment. The estimation of the genetic correlation between Y and E complements the correlation test between the PGI and the environment in the analysis sample, because non-significance of the latter correlation may be caused by differences between the discovery and analysis sample. Moreover, the absence of a genetic correlation between Y and E would allow for a G×E analysis despite a significant correlation between the PGI and E. The test we propose is of complementary value because if GWAS weights are proportional to true SNP effects, only then $Corr\left( {PGI_{Y} ,\,E_{g} } \right) = r_{g} \left( {Y,\,E} \right)$, where $E_{g}$ is the genetic component of the environment E. However, GWAS weights are not always proportional to the true SNP effects due to measurement error (Van Kippersluis et al. 2023), shrinkage due to LD regularization (Vilhjálmsson et al. 2015), GWAS model specification (Mostafavi et al. 2020), and sample selection (Van Alten et al. 2024). A simple correlation test between the PGI and the environment may appear to be zero, since the test is unable to always to capture such cases, so we would have $Corr\left( {PGI_{Y} ,\,E_{g} } \right)=0$, while $r_{g} \left( {Y,\,E} \right)> 0$ (see Appendix A for formal derivations). Moreover, in practice, $Corr\left( {PGI_{Y} ,\,E_{g} } \right)$ is not typically studied because it is not always possible to construct a PGI for the environment. Typically, E’s are very specifically defined in G×E studies and not generally available in GWAS training samples. GREML does not estimate SNP effects but assumes that these effects originate from a normal distribution, and by estimating the SNP-based heritability of the environment in the G×E analysis sample, orthogonality of the environmental condition to genetic differences can be assessed. Appendix B illustrates these insights using simulations.

If $Corr\left( {PGI_{Y} ,\,E_{g} } \right) = 0$, while $r_{g} \left( {Y,\,E} \right)> 0$, gene-environment interaction estimates are not necessarily biased because $r_{g} \left( {Y,\,E} \right)$ can result from an effect of E on Y. However, $r_{g} \left( {Y,\,E} \right)> 0$ is a more precise signal for potential bias than $Corr\left( {PGI_{Y} ,\,E_{g} } \right)> 0$, and helps us understand the mechanisms behind the G×E interaction better. Moreover, if $Corr\left( {PGI_{Y} ,\,E_{g} } \right)> 0$, the bivariate GREML test provides additional insights on the mechanisms contributing to this non-zero correlation between the PGI and the environment. For example, $r_{g} \left( {Y,\,E} \right) = 0$ when $Corr\left( {PGI_{Y} ,\,E_{g} } \right)> 0$ helps rule out genetic channels behind the positive gene-environment correlation and turn the analysis towards environmental causes, including among others, non-genetic selection, or differential distributions of the environmental condition in the GWAS training and analysis sample. In case a PGI constructed for a different trait X (e.g., educational attainment) is used to analyze outcome Y (e.g., ADHD) in the G×E analysis sample, finding $r_{g} \left( {Y,\,E} \right) > 0$when $Corr\left( {PGI_{X} ,\,E_{g} } \right) = 0$ points to genetic mechanisms not captured in $\:{PGI}_{X}$ potentially explaining the interaction.

## Methods

### Sample

Using genetic and phenotypic data from the UK Biobank (UKB) sample (Bycroft et al. 2018), we test for the orthogonality of the genetic propensity for educational attainment and three environmental conditions that are widely used in the G×E literature. UKB provides genetic and socio-economic information for approximately 500,000 volunteering and consented individuals (Fry et al. 2017). Participation in the UK Biobank is voluntary; hence, it is not a representative sample of the UK population. The UKB cohort consists of an adult population, 40 to 70 years old between 2006 and 2010. In the analysis sample, we only include European ancestry respondents (88%), who were identified on the basis of a self-report and a score of ≤ 0 on the first principal component of the genetic relatedness matrix (provided by UKB). We split the UKB into a discovery sample for GWAS analysis, and a genetically unrelated analysis sample in which the GWAS results are used to construct a polygenic index.

As environmental conditions, we analyze the RoSLA 1972 reform, gender, and birth district social class. To select our analysis sample, the RoSLA reform is leading. Following earlier studies (Barcellos et al. 2018), we select all individuals in the UK Biobank sample who were most eligible to be affected by the reform, i.e., individuals born up to two years before and after September 1957 (i.e., between September 1955 and August 1959). For the analyses with gender and birth district social class, we use the same set of individuals. Besides participants without information about their years of education, we also removed participants reporting an age at completing full-time education below the minimum school leave age from the analysis sample. The resulting G×E analysis sample comprises 36,444 individuals.

The GWAS sample is constructed by excluding individuals born after 1952 and who do not pass quality control filters. More specifically, we exclude individuals who have missing gender information or whose self-reported gender does not match the genetic sex, are of other than European ancestry, have poor genotyping quality, have putative sex chromosome aneuploidy, whose second chromosome karyotypes are different from XX or XY, with outliers in heterozygosity, or have missing information on any of the former criteria. We also drop those who withdrew consent over the years. Excluding those born after 1952 allows us to have a non-overlapping window of 2 year before and after the cohort affected by RoSLA1972 (1955–1959) in our holdout sample for PGI-based analyses. The cohort born after 1959 is also excluded from the GWAS discovery sample because they were already affected by RoSLA 1972. The GWAS discovery sample comprises 246,542 individuals.

### Measures

To construct the dependent variable, we converted individuals’ highest earned qualifications to equivalent years of education using the International Standard Classification of Education (ISCED), following the literature (Rietveld et al. 2013; Okbay et al. 2016; Lee et al. 2018). Specifically, years of education ranges from 7 to 20, where College or University degree is equivalent to 20 years, National Vocational Qualification (NVQ), Higher National Diploma (HND), or Higher National Certificate (HNC) to 19 years, other professional qualifications to 15 years, having an A or AS levels similar to 13 years, O levels, (General) Certificate of Secondary Education ((G)CSE) to 10 years, and if none of the above to the lowest level of 7 years.

We consider three environmental conditions. The first condition is the Raising of School Leave Age of 1972. It is operationalized with a binary indicator being equal to 1 for all UKB participants born on and after September 1, 1957, and 0 otherwise. The second condition is gender, defined as a binary indicator equal to 0 for females and 1 for males The third condition reflects the birth district socio-economic status (SES). This binary variable is equal to 1 if the birth district has an above median proportion of individuals in high social classes (i.e. Professional/Managerial/Technical backgrounds), and 0 otherwise. The latter variable is constructed using data from the 1951 UK Census on the socio-economic composition of individuals’ Local Government of birth. The results are qualitatively the same when using an alternative available measure of SES. Specifically, they are qualitatively the same when we categorize the districts of birth into high-SES (with below median proportion of males who left school at age 15 in the Census) and low-SES (with above median proportion of males who left school at age 15 in the Census).

### Statistical Analysis

For GWAS analysis, we use the fastGWA tool for Genome-wide Complex Trait Analysis (Jiang et al. 2019), which applies mixed linear modelling (MLM) to the genetic data. Using fastGWA requires the following steps. First, we generate a sparse genetic relatedness matrix (GRM) based on the family relatedness provided by the UKB. Next, we perform an MLM-based GWAS using the SNP data, the sparse GRM, our phenotype of interest (educational attainment) and the minor allele frequency (MAF) filter of 0.001. The phenotype is residualised with respect to birth year dummies, gender, interaction of birth year and gender, batch, and the first 40 principal components (PCs) of the genetic relatedness matrix. We apply quality control to the resulting GWAS summary statistics using EasyQC (Winkler et al. 2014).

To maintain independence of the samples, we removed individuals from the analysis sample related to individuals in the GWAS sample. These individuals are identified using the UKB’s kinship matrix which is based on genetic relatedness and contains relatives of third degree and closer. This matrix is computed using the KING software (Manichaikul et al. 2010). The UK Biobank does not have information on self-reported relatedness (Bycroft et al. 2018). The degree of relatedness between the pairs of individuals is based on the combination of the kinship coefficient and genetic similarity in terms of the identity by state (IBS_0_) coefficient. IBS_0_ measures the fraction of markers for which the related individuals do not share alleles. We follow the KING manual for the thresholds on classifying the family relationships. This ensures that our holdout sample for PGI construction and prediction is unrelated to the GWAS discovery sample which is used to calibrate the SNP weights and construct the polygenic indices. In the holdout sample, the PGI has been standardized to have mean 0 and standard deviation of 1. Due to missing data on location at birth, the number of observations is lower in the birth district social class analyses.

Construction of the polygenic index involved two steps. First, we adjusted the GWAS summary statistics for linkage disequilibrium (LD). Using LDpred (version 1.06) (Vilhjálmsson et al. 2015; Privé et al. 2020), we retained all available HapMap3 SNPs (1,173,308) that passed quality control and used the correlation structure of these SNPs in a Bayesian approach to adjust the GWAS coefficients for LD. A random sample of 30,000 unrelated UKB participants served as the reference panel for LD calculations. We assumed the proportion of causal SNPs to be 1, as is standard practice for highly polygenic traits (Becker et al. 2021). We used PLINK (Purcell et al. 2007) to build a polygenic index with the re-weighted SNP effects.

To compare the distribution of the PGI across the environmental strata, we use three different tests. The first one is the well-known *t*-test, which tests if there are differences in the mean of the PGI distribution across two environmental strata. Effectively, in case of two environmental strata, this is a simple test on the correlation between two variables. By residualizing the PGI for the set of covariates, we also look at the conditional correlation between these residuals and the environmental conditions. Whereas the *t*-test and conditional correlation only compare the difference in means, our third test, the Kolmogorov-Smirnov (KS) test (Massey 1951), takes into consideration any difference in the PGI distribution across the two samples. This means that it is more versatile compared to the previous tests. While not our main focus, we include this test because GxE studies often compare the PGI distribution across environmental strata (e.g., Muslimova et al. 2024). In case of a (conditional) difference in means, both the *t*-test, conditional correlation, and the KS test will detect this. However, if there is a difference in the variances or skews, a *t*-test and conditional correlation might not pick this up whereas the KS test will. If the distributions are different, this could indicate that the environment is not orthogonal to genetic differences. There are also alternative tests available for distribution comparison, such as the K-sample Anderson–Darling and Cramér–von Mises statistics, however these methods are not as readily available in statistical software as the *t*-test, conditional correlation, and KS test and the results tend to be quite similar to those from the KS test.

By testing for a genetic correlation, it is possible to analyze to what extent two outcomes are genetically intertwined. In this study, we employ bivariate-GREML as implemented in MGREML (De Vlaming et al. 2021) because it does not require GWAS summary statistics as input (like, for instance, LD Score regression; Bullik-Sullivan et al. 2015). GREML estimates what part of the variance in a trait can be explained by all common SNPs ($\:{h}_{g}^{2}$). Bivariate GREML estimates the quantity $\:{h}_{g}^{2}$ for two traits and estimates the genetic correlation ($\:{\rho\:}_{g}$) between these two traits (Lee et al. 2012).

## Results

Table 1 provides the descriptive statistics for the analysis sample used in the empirical application. In the analysis sample, the average years of education, our main variable of interest, is 16 years. About 49% of the participants are affected by the raising of school leave age reform and about 44% of the participants are male. Roughly 28% of the participants had an above median proportion of high SES individuals in their birth district. The average birth year is 1957, with a standard deviation of 1.15.

**Table 1:** Descriptive statistics analysis sample (*N* = 36,444)

	Mean	Standard deviation	Minimum	Maximum
Educational attainment	15.95	4.64	7	20
Polygenic index of educational attainment	0.00	1.00	−3.88	4.09
RoSLA	0.49	0.50	0	1
Gender (1 = Male; 0 = Female)	0.44	0.50	0	1
Birth district social class (1 = above median, 0 = below median)	0.28	0.45	0	1
Birth year	1957.69	1.15	1955.75	1959.67

RoSLA is a binary indicator equal to 1 (0) if the individual is born after (before) September 1, 1957. Gender is coded as 1 (0) for males (females). Birth district social class is a binary indicator equal to 1 (0) if the individual’s birth district has an above (below) median proportion of individuals in higher social classes (*N* = 31,458)

**Fig. 1:**
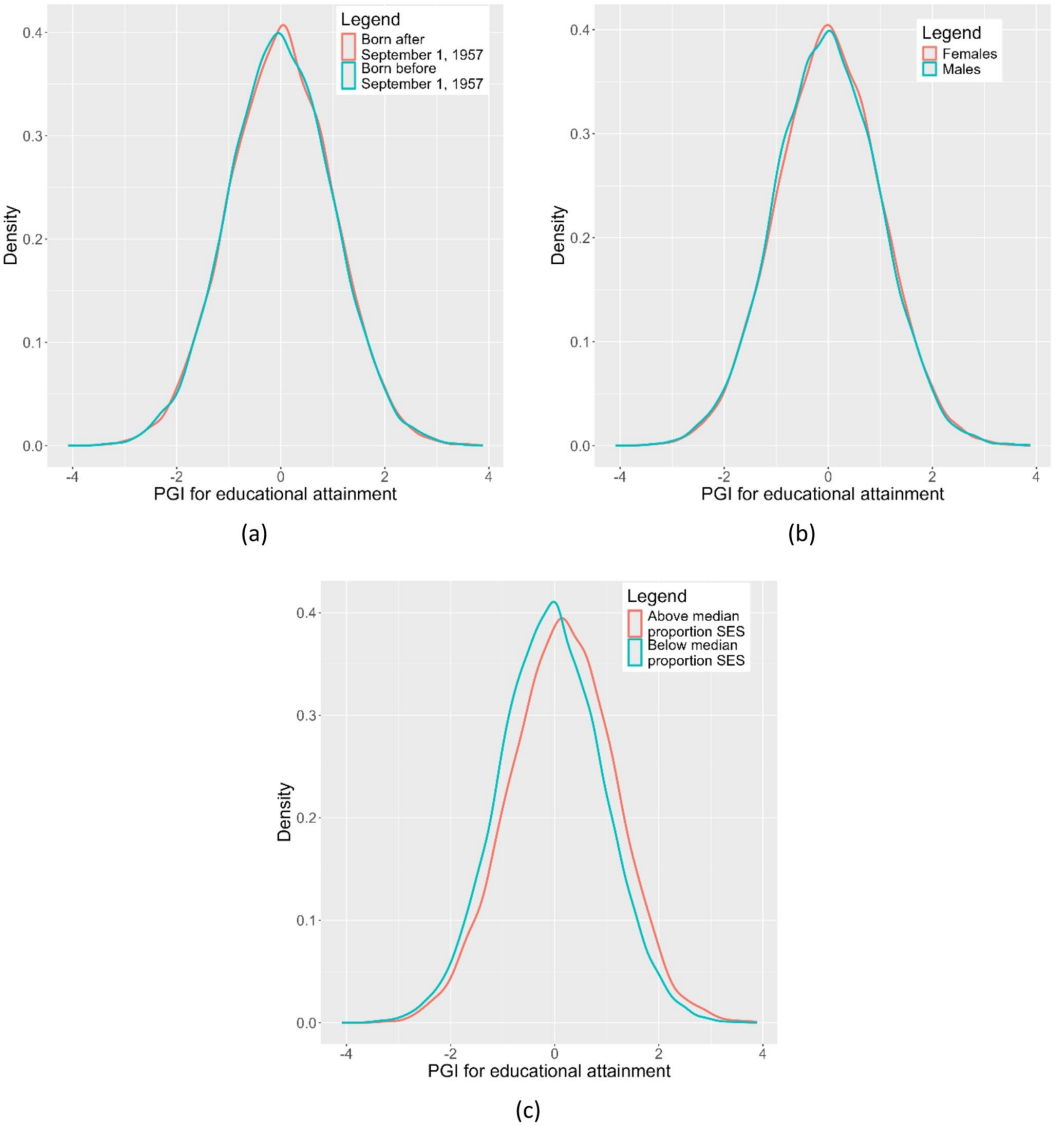
The distribution of the polygenic index (PGI) of educational attainment (EA) stratified by three environmental exposures (Raising of School Leave Age (RoSLA) 1972, Gender, Birth district social class). **a** Raising of School Leave Age, (1972), **b** Gender, **c** Birth district social class

Figure 1 displays the distribution of the PGI across the different environmental strata. Table 2 includes the main empirical results. The *t*-tests on the observed correlation between the PGI and environments reveal a statistically significant difference in the PGI distribution with respect to gender and birth district social class. These relations between PGI distribution and gender and birth district remain significant even after correcting for the set of typical controls, as is indicated by the conditional correlation. Kolmogorov-Smirnov tests confirm these results by comparing more flexibly the differences in the shape of the PGI distribution across environmental strata. With the GWAS sample and G×E analysis sample both stemming from UK Biobank, and thus being genetically more similar than typical in PGI-based G×E analyses, these differences are noteworthy.

The SNP-based heritabilities of educational attainment and the environmental exposures are obtained using GREML estimation. In our analyses, we control for the first 40 principal components of the genetic relatedness matrix. In the three (overlapping) samples, the SNP-based heritability of educational attainment varies between 18.5% and 20.7%. With the SNP-based heritability of RoSLA being a clean zero, its genetic correlation with educational attainment appears uninformative. However, both the SNP-based heritability of gender and birth district social class are significantly different from zero, respectively 3.3% and 15.6%. While the genetic correlation between gender and educational attainment is not statistically distinguishable from zero, it is 0.368 (SE 0.052) between birth district social class and educational attainment.

The results of these tests imply that the genetic propensity for educational attainment is orthogonal to both the RoSLA 1972 reform and gender, such that these environmental exposures can be used for G×E interplay analysis. For the RoSLA 1972 reform, there is no statistically significant difference in the PGI distribution across the environmental strata nor a genetic overlap between them. With respect to gender, there is some difference in the PGI distribution, but it appears that there is no genetic overlap between educational attainment and this environmental exposure. However, educational attainment and birth district social class appear to be genetically intertwined. This is evident from the means-based test as well as the genetic correlation. The lack of orthogonality implies that birth district social class cannot be used as an exogenous moderator in G×E models because the direction of the bias in the estimated interaction effect is undetermined (Pasman et al. 2022).

**Table 2:** Results of statistical tests assessing the orthogonality of the genetic propensity for educational attainment (EA) and three environmental conditions (Raising of school leave age (RoSLA) 1972, Gender, birth district social class)

	Raising of School Leave Age (1972)	Gender	Birth district social class
$\rho _{{PGI_{{EA}} ,Env.}}$	0.004	−0.0133	0.099
*t*-statistic	0.722	−2.533	17.572
*p*-value	0.470	0.011	8.58 × 10^− 69^
$\rho _{{PGI_{{EA}} ,Env. | Covariates}}$	0.004	−0.011	0.090
*p*-value	0.448	0.029	1.98 × 10^− 57^
*KS*-statistic	0.009	0.015	0.098
*p*-value	0.415	0.025	< 2.20 × 10^− 16^
$h_{{SNP}}^{2}$ (EA)	0.185 (0.011)	0.185 (0.011)	0.207 (0.022)
$h_{{SNP}}^{2}$ (Env.)	0.000 (0.009)	0.033 (0.010)	0.156 (0.022)
$\rho _{g}$	N.A.	−0.010 (0.096)	0.368 (0.052)
*p*-value	N.A.	0.915	1.02 × 10^− 12^
*N*	36,444	36,444	31,458

$\rho _{{PGI_{{EA,}} Env .}}$ refers to the correlation between the polygenic index (PGI) for educational attainment (EA) and the environment; the *t*-statistic assesses the difference in means of the distribution of the PGI between environmental strata; the *p*-value corresponding to $\rho _{{PGI_{{EA,}} Env.}}$ and the *t*-statistic are the same; $\rho _{{PGI_{{EA}} ,Env. | Covariates}}$ refers to the correlation between the PGI and the environment after residualizing the PGI for the relevant covariates (40 leading principal components plus i) gender for the RoSLA1972 analysis; ii) year of birth and month of birth for the gender and birth district social class analyses); The Kolmogorov-Smirnov (*KS*)-statistic assesses the difference in shape of the distribution between environmental strata; $h_{{SNP}}^{2}$ (EA) and $h_{{SNP}}^{2}$ (Env.) are the SNP-based heritabilities of educational attainment (EA) and the environmental condition (Env.), with standard errors in parentheses; $\rho _{g}$ is the genetic correlation between EA and the environmental condition, with standard errors in parentheses; The corresponding *p*-value is the result of the likelihood ratio test $\rho _{g} = 0$; *N* is the number of individuals in the analysis sample after excluding genetically related individuals using a 0.025 threshold. All results are obtained in the G×E analysis sample (i.e., not in the GWAS training sample). Due to estimating $h_{{SNP}}^{2}$ (Env.) = 0.000 for RoSLA1972, $\rho _{g}$ is not available for this outcome

Since birth district social class and educational attainment are not orthogonal, we only report G×E analyses for RoSLA and gender. The results are available in Table 3. Following the recommendations by Keller (2014), we also report the results of a robust specification that controls for interactions between the environment and control variables. The results suggest that a 1 standard deviation in PGI for educational attainment increases educational attainment by about 1.1 years. RoSLA increases educational attainment by 0.2 year and gender by roughly 0.37 year. The interaction term PGI_EA_ × Environment is not statistically significant in any specification, which might be attributable to the relatively small sample size. The (statistically insignificant) coefficient for gender is very large in the robust specification because of the inclusion of many correlated covariates.

**Table 3:** Results of G×E analyses on educational attainment with Raising of the school leave age 1972 (RoSLA) and gender as environmental conditions

	Simple specification		Robust specification	
	RoSLA	Gender	RoSLA	Gender
Main variables				
PGI_EA_	1.113***	1.066***	1.111***	1.067***
	(0.033)	(0.031)	(0.033)	(0.032)
Environment	0.206***	0.374***	0.103	−75.456
	(0.047)	(0.047)	(0.383)	(81.084)
PGI_EA_ × Environment	−0.081	0.017	−0.079	0.012
	(0.047)	(0.047)	(0.047)	(0.048)
Covariates				
Gender	✔		✔	
40 PCs	✔	✔	✔	✔
Month of birth		✔		✔
Year of birth		✔		✔
PGI_EA_^2^			✔	✔
Gender × Environment			✔	
40 PCs × Environment			✔	✔
Month of birth × Environment				✔
Year of birth × Environment				✔

PGI_EA_ is the polygenic index for educational attainment. RoSLA is a binary indicator equal to 1 (0) if the individual is born after (before) September 1, 1957. Gender is a binary indicator equal to 1 (0) if the individual is male (female). PCs are the principal components of the genetic relatedness matrix. ***P < 0.01, **P < 0.05,*P < 0.10

## Discussion

Accurate estimates of gene-environment interactions are important to advance our understanding of how nature and nurture interact in shaping individual outcomes (Miao et al. 2025). Contributing to the literature on the validity of PGI-based gene-environment interaction studies (Keller 2014; Domingue et al. 2020; Biroli et al. 2025), we considered the orthogonality of genetic propensities and environmental conditions. We argue for the need to complement the typical correlation-based test with an assessment of the genetic correlation between outcome Y and environment E to assess this orthogonality. While our tests confirm the exogeneity of the RoSLA 1972 reform and gender, birth district social class does not pass either of the tests. Although nowadays pre-constructed PGIs are increasingly available in datasets (Becker et al. 2021; Alemu et al. 2025), we show that it is important to not solely rely on these genetic summary indices when performing G×E analyses. As such, it remains important to keep training applied researchers to work with SNP-level genetic data.

## Supplementary Information

Below is the link to the electronic supplementary material.

Supplementary Material 1

## Data Availability

Our study is partly based on data from the UK Biobank. These data are only available upon registration, such that we cannot make the data publicly available.
