# Supplementary material for: Assessing Orthogonality in Gene-Environment Interaction Studies Using Polygenic Indices
Source: Behav Genet. 2026 Jan 9;56(1):39–47. doi: 10.1007/s10519-025-10248-8 (PMC12795928; doi:10.1007/s10519-025-10248-8)
Supplement: Supplementary file 1 — Supplementary Material 1 [file 10519_2025_10248_MOESM1_ESM.pdf]

Appendix

Assessing orthogonality in gene-environment interaction studies using polygenic indices

Eric A.W. Slob, Dilnoza Muslimova, and Cornelius A. Rietveld

Corresponding author: Eric A.W. Slob

Email: e.slob@essb.eur.nl

A. The correlation-based test vs. bivariate GREML

Corr(PGI$_Y$, E). Let $G \in \mathbb{R}^{n \times m}$ be the matrix of standardized genotypes (rows = individuals $i = 1, \dots, n$; columns = SNPs $j = 1, \dots, m$). The population level LD matrix is:

$$S = \frac{1}{n}\mathbb{E}[G'G] \in \mathbb{R}^{m \times m}.$$

Let w be the fixed PGI_Y weights from a GWAS for outcome Y, so $w \in \mathbb{R}^m$ and $PGI_Y = Gw$. We also introduce the additive genetic component of the environment E is $E_g = G\gamma$ with $\gamma \in \mathbb{R}^m$,so the observed E can be expresses as $E = E_g + \eta_E$ with $\mathbb{E}[G'\eta_E] = 0$. Unless noted otherwise, w and γ are treated as fixed vectors.

Let:

$$Cov(PGI_Y, E) = w'S\gamma, \qquad Var(PGI_Y) = w'Sw, \qquad Var(E) = \gamma'S\gamma + Var(\eta_E).$$

Hence, the correlation between the PGI_Y and the observed E can be formalized as follows:

$$Corr(PGI_Y, E) = \frac{w'S\gamma}{\sqrt{(w'Sw)\big(\gamma'S\gamma + Var(\eta_E)\big)}},$$

and the genetic component of the correlation as follows:

$$Corr\big(PGI_Y, E_g\big) = \frac{w'S\gamma}{\sqrt{(w'Sw)(\gamma'S\gamma)}}.$$

Genetic correlation, $r_g(Y, E)$. Let $K = \frac{1}{m}GG' \in \mathbb{R}^{n \times n}$ be the genomic relationship matrix (GRM) and I_n the $n \times n$ identity matrix. The genetic correlation obtained using the bivariate GREML approach could be formalized as follows:

$$Y = u_Y + e_Y, \qquad u_Y \sim \mathcal{N}\big(0, \sigma^2_{gY}K\big), \qquad e_Y \sim \mathcal{N}\big(0, \sigma^2_{eY}I\big),$$

$$E = u_E + e_E, \qquad u_E \sim \mathcal{N}\big(0, \sigma^2_{gE}K\big), \qquad e_E \sim \mathcal{N}\big(0, \sigma^2_{eE}I\big),$$

$$Cov(u_Y, u_E) = \sigma_{g,YE}K \text{ and } Cov(e_Y, e_E) = 0.$$

GREML estimates the following variance components: σ^2_{gY} – additive genetic variance of Y, σ^2_{gE} – additive genetic variance of E, $\sigma_{g,YE}$ – additive genetic covariance between Y and E, and σ^2_{eY} – residual variance of Y, σ^2_{eE} –residual variance of E. From these components the covariances and genetic correlation can be obtained as follows:

$$Cov_g(Y, E) = \sigma_{g,YE}, \qquad r_g(Y, E) = \frac{\sigma_{g,YE}}{\sqrt{\sigma^2_{gY}, \sigma^2_{gE}}}.$$

Under the standard "many small effects" approximation for complex standardized traits, let $\beta \in \mathbb{R}^m$ denote the fixed vector for the additive genetic component of the outcome Y in the target population (so $G\beta$ is the additive component of Y). Note, that β is typically distinct from GWAS weights, w. Then approximately:

$$\sigma^2_{gY} \approx \beta'S\beta,$$

$$\sigma^2_{gE} \approx \gamma'S\gamma,$$

$$\sigma_{g,YE} \approx \beta' S\gamma,$$

and therefore

$$r_g(Y,E) \approx \frac{\beta' S\gamma}{\sqrt{(\beta' S\beta)(\gamma' S\gamma)}}.$$

If GWAS weights are proportional to the true SNP effects, that is $w = c\beta$ for some constant c, then $Corr(PGI_Y, E_g) = r_g(Y,E)$. Interestingly, given that the variance of the observed E is $Var(E) = \gamma' S\gamma + Var(\eta_E)$ and the SNP-based heritability of E is $h_E^2 = \frac{\gamma' S\gamma}{Var(E)}$, in case $w = c\beta$, we obtain:

$$\begin{aligned}
Corr(PGI,E) &= \frac{w' S\gamma}{\sqrt{(w' Sw)Var(E)}} \\
&= \frac{c\beta' S\gamma}{\sqrt{(c^2\beta' S\beta)Var(E)}} \\
&= \frac{c\beta' S\gamma}{c\sqrt{(\beta' S\beta)}Var(E)} \frac{\sqrt{\gamma' S\gamma}}{\sqrt{\gamma' S\gamma}} \\
&= \frac{c\beta' S\gamma}{c\sqrt{(\beta' S\beta)}\sqrt{\gamma' S\gamma}} \sqrt{\frac{\gamma' S\gamma}{Var(E)}} \\
&= r_g(Y,E)\sqrt{h_E^2}.
\end{aligned}$$

Because $h_E^2 \in [0,1]$, it follows that $\sqrt{h_E^2} \in [0,1]$. Therefore,

$$|Corr(PGI,E)| \leq |r_g(Y,E)|.$$

In practice, $w \neq c\beta$ due to measurement error, shrinkage due to regularization and LD corrections, differences between discovery and analysis sample, due to distribution of the environment, phenotype definition, model specifications, and selection. In this case, $w' S\gamma$ can be ≈ 0, yielding $Corr(PGI_Y, E)$ ≈ 0 while $\beta' S\gamma > 0$ (i.e., $r_g(Y,E) > 0$). To exemplify, GWAS weights w obtained from one (meta-analytic) sample may turn out to be a poor proxy for β in the analysis sample, potentially leading to biased $G \times E$ estimates.

In Appendix Figure 1, we present a Directed Acyclic Graph (DAG) visualizing the difference and similarity between the correlation-based test between PGI_Y and E, and the genetic correlation between Y and E as estimated using bivariate GREML. The GWAS sample and the G×E analysis sample are independent, as they contain non-overlapping sets of participants. In the GWAS sample, we estimate the weights w to construct the PGI of outcome Y in the analysis sample. The correlation-based test can be thought of as a standardized measure of how strongly the PGI constructed using these weights w and the environment E covary in the analysis sample environment. Note that this takes into account both the genetic and non-genetic contribution to E. Next, bivariate GREML quantifies the extent the genetic component of Y covaries with the genetic component of E in the analysis sample.

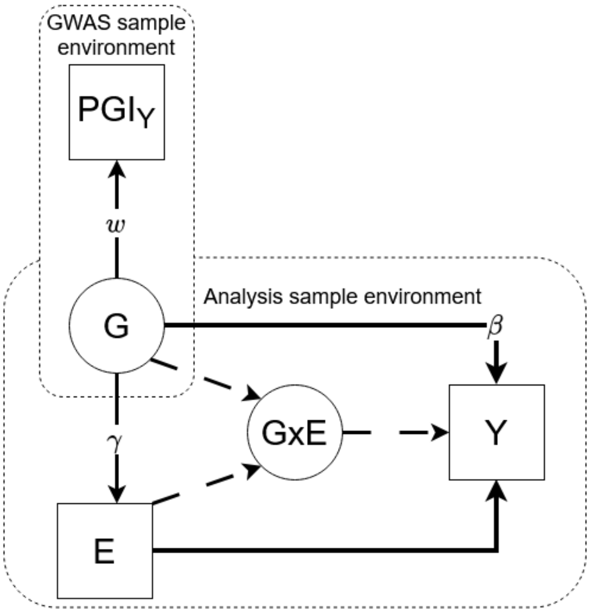

Appendix Figure 1. Directed Acyclic Graph (DAG) visualizing the difference and similarity between the correlation-based test between the polygenic index (PGI) of outcome *Y* and *E*, and the genetic correlation between *Y* and *E* as estimated using bivariate GREML.

B. Simulations to assess possible bias in G×E analyses

To assess possible bias a typical G×E analysis, we simulate a typical two-sample analysis to study bias in gene–environment interaction (G×E) estimates. In each run, a discovery sample is used to perform a GWAS and generate SNP effect estimates, which are then used to construct a polygenic index (PGI) in an independent target sample. A binary environmental moderator ("RoSLA") is simulated to be heritable but genetically uncorrelated with the PGI, such that the PGI and RoSLA are orthogonal.

The outcome phenotype (y) is generated from main genetic effects, environmental effects, and true G×E interactions, with a parental PGI × environment term introducing bias. The data generating process for y is given by:

$$y = intercept\ +\ 1 * PGI\ +\ 1 * \ RoSLA + \ 1 * PGI\ X\ RoSLA\ +\ 1 * \ \left(PGI_{parental}\ *\ RoSLA\right) + \varepsilon,$$

where PGI corresponds to individuals polygenic score for outcome y, $RoSLA$ is the environment studied, $PGI_{parental}$ is the parental polygenic score for outcome y, and ε is a residual. Here, there is not only an interaction between RoSLA and the PGI, but also with RoSLA and the parental polygenic score. Since this parental polygenic score is correlated with the individual's polygenic score, this can create bias. The simulation code is provided in Appendix C.

Through a linear regression the G×E effect is estimated to assess bias across the simulations. We also estimate the genetic relationship matrices (GRM) for GREML estimation using GCTA (Yang et al., 2011), to assess the genetic correlation between RoSLA and the phenotype y. We ran the simulation 100 times. The results are shown in Appendix Table 1.

Appendix Table 1. Simulations result from 100 runs, where the mean of estimated parameters are shown, with corresponding estimated standard errors and empirical standard errors.

Parameter	Estimate	Estimated standard error	Empirical standard error
β_{PGI}	1.208	0.030	0.036
β_{RoSLA}	1.002	0.022	0.022
$\beta_{PGI \times RoSLA}$	1.191	0.042	0.064
$\rho(PGI, RoSLA)$	-0.001	0.012	0.012
p-value	0.497		
p-value<0.05	5.0%		
$\rho_G(y, RoSLA)$	0.132	0.073	0.065
p-value	0.089		
p-value<0.05	54.0%		

The simulations suggest that there is a substantial bias in the estimated G×E coefficient in the chosen setting (average estimated coefficient is 1.191, whereas the real effect is 1.000). The observed correlation between the PGI and RoSLA does not seem to be insightful in the case, as the rejection rate is equal to the type 1 error rate ($\alpha = 0.05$). The proposed genetic correlation check is significant in 54% of the simulations, which showcases that the check is relevant to perform.

C. *R* code for the simulations presented in Appendix B

```
#script to generate data where RoSLA and PGI are orthogonal phenotypically
, but genetically correlated
library(gaston)
library(broom)
set.seed(12345)
setwd("biasparentalpgi")
### Parameters
n_disc <- 10000
n_targ <- 7500
m <- 500
h2_true <- 0.35
prop_interaction <- 0.50
beta_r <- 1.0
intercept <- 0.0
gamma_par <- 0.5   # strength of the omitted C x RoSLA effect
runs=100
mOutputframe<-matrix(0,nrow=22,ncol=runs)
rownames(mOutputframe)<-c("cor(beta,gamma)","cor(PGI_targ, PRS_R)","cor(PG
I_targ, RoSLA_targ)","cor.test(PGI_targ, RoSLA_targ)$p.value","se(cor)","m
ean(RoSLA_targ)","beta_intercept","beta_PGI","beta_RoSLA","beta_PGIxRoSLA"
,"std_intercept","std_PGI","std_RoSLA","std_PGIxRoSLA","statistic_intercep
t","statistic_PGI","statistic_RoSLA","statistic_PGIxRoSLA","pval_intercept
","pval_PGI","pval_RoSLA","pval_PGIxRoSLA")
for (i in 1:runs){
  ### Simulate allele freqs & genotypes
  p <- runif(m, 0.05, 0.5)
  simulate_genotypes <- function(n, p) {
    mat <- sapply(p, function(pi) rbinom(n, 2, pi))
    return(as.matrix(mat))
  }
  G_disc <- simulate_genotypes(n_disc, p)
  G_targ <- simulate_genotypes(n_targ, p)

  ### Standardize using combined sample means/sds
  G_all <- rbind(G_disc, G_targ)
  G_mean <- colMeans(G_all)
  G_sd <- apply(G_all, 2, sd)
  G_sd[G_sd == 0] <- 1
  Gc_disc <- scale(G_disc, center = G_mean, scale = G_sd)
  Gc_targ <- scale(G_targ, center = G_mean, scale = G_sd)

  ### True SNP main and interaction effects (correlated)
  sum_beta_sq <- h2_true * (1 - prop_interaction)
  sum_gamma_sq <- h2_true * prop_interaction
  rho <- 0.6
  beta_raw <- rnorm(m)
  eps_raw  <- rnorm(m)
  gamma_raw <- rho * beta_raw + sqrt(1 - rho^2) * eps_raw
  # scale to allocate variance
  beta <- beta_raw / sqrt(sum(beta_raw^2)) * sqrt(sum_beta_sq)
  gamma <- gamma_raw / sqrt(sum(gamma_raw^2)) * sqrt(sum_gamma_sq)
  mOutputframe[1,i]=cor(beta,gamma)
```

```r
  ### Discovery outcome (RoSLA = 0 for everyone)
  RoSLA_disc <- rep(0, n_disc)
  Gscore_disc <- as.vector(Gc_disc %*% beta)
  Gx_disc <- as.vector(Gc_disc %*% gamma) * RoSLA_disc
  var_g_main <- var(Gscore_disc)
  sigma_e2 <- 1 - var_g_main
  if (sigma_e2 <= 0) stop("Negative or zero residual variance; adjust parameters.")
  eps_disc <- rnorm(n_disc, 0, sqrt(sigma_e2))
  y_disc <- intercept + Gscore_disc + beta_r * RoSLA_disc + Gx_disc + eps_disc

  ### GWAS: regress on standardized SNPs (so betas live in standardized SNP-space)
  betas_gwas <- numeric(m)
  for (j in 1:m) {
    fit <- lm(y_disc ~ Gc_disc[, j])
    betas_gwas[j] <- coef(fit)[2]
  }
  # discovery weights on standardized scale
  w_raw <- betas_gwas

  ### Target: PGI computed on standardized target genotypes (consistent scale)
  PGI_targ <- as.vector(Gc_targ %*% w_raw)

  ### Construct a heritable RoSLA whose genetic liability is orthogonal to PGI_targ
  # 1) choose raw delta and compute S = t(Z) %*% Z (target standardized genotypes)
  Z <- Gc_targ            # n_targ x m standardized
  S <- crossprod(Z)       # m x m matrix

  # 2) draw raw delta
  delta_raw <- rnorm(m)

  # 3) compute scalar c that removes component correlated with w_raw under S
  num <- as.numeric(crossprod(w_raw, S %*% delta_raw))
  den <- as.numeric(crossprod(w_raw, S %*% w_raw))
  if (abs(den) < 1e-12) stop("Denominator too small for orthogonalization; check w_raw.")
  c_scalar <- num / den

  # 4) orthogonalize delta
  delta <- delta_raw - c_scalar * w_raw

  # 5) scale delta so PRS_R has desired liability heritability
  # choose desired liability-scale h2 for RoSLA (on latent scale)
  h2_R_liab <- 0.20   # change as desired
  PRS_R_raw <- as.vector(Z %*% delta)
  # scale to desired variance of genetic liability
  PRS_R <- PRS_R_raw * sqrt(h2_R_liab / var(PRS_R_raw))
```

```r
  # 6) generate binary RoSLA via logistic (approx 50% prevalence)
  # choose intercept alpha to get ~0.5 prevalence; because PRS_R is mean ~
0 use alpha=0
  alpha <- 0
  p_R <- plogis(alpha + PRS_R)
  RoSLA_targ <- rbinom(n_targ, 1, p_R)

  # 7) quick checks
  cor_pgi_prsR <- cor(PGI_targ, PRS_R)
  objcor<-cor.test(PGI_targ, RoSLA_targ)
  secor<-unname(sqrt((1 - objcor$estimate^2)/objcor$parameter))
  #save correlation
  mOutputframe[2,i]=cor(PGI_targ, PRS_R)
  mOutputframe[3,i]=cor(PGI_targ, RoSLA_targ)
  mOutputframe[4,i]=cor.test(PGI_targ, RoSLA_targ)$p.value
  mOutputframe[5,i]=secor
  mOutputframe[6,i]=mean(RoSLA_targ)
  # 8) Build phenotype in target (with added parental pgi x rosla interact
ion to get bias in gxe estimate)

  parentalpgiresid <- rnorm(m)
  # make kappa correlate with w_raw by mixing
  corr_strength <- 0.5
  kappa <- corr_strength * (w_raw / sqrt(sum(w_raw^2))) + sqrt(1 - corr_st
rength^2) * (parentalpgiresid / sqrt(sum(parentalpgiresid^2)))
  parentalpgi_raw <- as.vector(Gc_targ %*% kappa)
  parentalpgi <- scale(parentalpgi_raw)[,1]   # standardize

  Gscore_targ <- as.vector(Gc_targ %*% beta)
  Gx_targ <- as.vector(Gc_targ %*% gamma) * RoSLA_targ
  eps_targ <- rnorm(n_targ, 0, sqrt(sigma_e2))
  y_targ <- intercept + Gscore_targ + beta_r * RoSLA_targ + Gx_targ + eps_
targ +gamma_par * (parentalpgi * RoSLA_targ)

  #run GxE regression
  regressionmodel<-lm("y_targ~Gscore_targ + RoSLA_targ + Gx_targ")
  out<-tidy(regressionmodel)
  mOutputframe[7:10,i]=out$estimate
  mOutputframe[11:14,i]=out$std.error
  mOutputframe[15:18,i]=out$statistic
  mOutputframe[19:22,i]=out$p.value
  # 9) GRM and GREML
  GRM_mat <- tcrossprod(Z) / m
  GRM_mat <- (GRM_mat + t(GRM_mat)) / 2

  #### -----------------------------------------------------------
  ### EXPORT for GCTA (GRM in binary format + phenotype file)
  ### -----------------------------------------------------------

  # Set a prefix for output files
  prefix <- paste("simulation_target",i,sep="_")
  # # 1. GRM export (binary format)  GCTA expects .grm.bin, .grm.N.bin, .g
rm.id
  n_targ <- nrow(GRM_mat)
```

```
  m_targ <- ncol(Z)  # number of SNPs

  # (a) ID file
  ID <- data.frame(FID = 1:n_targ, IID = 1:n_targ)
  write.table(ID,
              paste0(prefix, ".grm.id"),
              quote = FALSE, row.names = FALSE, col.names = FALSE)

  # (b) GRM binary (upper triangle including diagonal)
  #     GCTA stores as 32-bit doubles (size=4)
  #     Ensure symmetry and double precision
  GRM_mat <- (GRM_mat + t(GRM_mat)) / 2
  grm_vals <- GRM_mat[upper.tri(GRM_mat, diag = TRUE)]
  writeBin(as.numeric(grm_vals), paste0(prefix, ".grm.bin"), size = 4)
  # (c) Number of SNPs per pair (here constant m)
  #     Store in the same order as upper triangle
  N_vals <- rep(m_targ, length(grm_vals))
  writeBin(as.integer(N_vals), paste0(prefix, ".grm.N.bin"), size = 4)

  # 2. Phenotype export: y_targ and RoSLA_targ
  pheno <- data.frame(FID = 1:n_targ,
                      IID = 1:n_targ,
                      y_targ = y_targ,
                      RoSLA_targ = RoSLA_targ)
  write.table(pheno,
             paste0(prefix, ".phenotypes.txt"),
             quote = FALSE, row.names = FALSE, col.names = TRUE, sep = "\t
")


}
# write away outputframe
write.csv(mOutputframe,'mOutputframe100runs.csv',row.names=TRUE)
# create batch file to run univariate and bivariate GREML
sink("gctacommand.bat")
for (i in 1:runs){
  cat(paste("gcta64 --grm simulation_target_",i," --pheno simulation_targe
t_",i,".phenotypes.txt --mpheno 1 --reml --out simulation_target_",i,"_phe
no1_out",sep=""))
  cat("\n")
  cat(paste("gcta64 --grm simulation_target_",i," --pheno simulation_targe
t_",i,".phenotypes.txt --mpheno 2 --reml --out simulation_target_",i,"_phe
no2_out",sep=""))
  cat("\n")
  cat(paste("gcta64 --grm simulation_target_",i," --pheno simulation_targe
t_",i,".phenotypes.txt --reml-bivar --reml-bivar-lrt-rg 0 --out simulation
_target_",i,"_bivar_out",sep=""))
  cat("\n")
}
sink()
```
